# Prioritization of biological processes based on the reconstruction and analysis of associative gene networks describing the response of plants to adverse environmental factors

**DOI:** 10.18699/VJ21.065

**Published:** 2021-09

**Authors:** P.S. Demenkov, Е.А. Oshchepkova, P.S. Demenkov, T.V. Ivanisenko, V.A. Ivanisenko

**Affiliations:** Institute of Cytology and Genetics of the Siberian Branch of the Russian Academy of Sciences, Novosibirsk, Russia Novosibirsk State University, Novosibirsk, Russia; Institute of Cytology and Genetics of the Siberian Branch of the Russian Academy of Sciences, Novosibirsk, Russia; Institute of Cytology and Genetics of the Siberian Branch of the Russian Academy of Sciences, Novosibirsk, Russia Novosibirsk State University, Novosibirsk, Russia; Institute of Cytology and Genetics of the Siberian Branch of the Russian Academy of Sciences, Novosibirsk, Russia; Novosibirsk State University, Novosibirsk, Russiavosibirsk, Russia Kurchatov Genomic Center of ICG SB RAS, Novosibirsk, Russia

**Keywords:** knowledge base SOLANUM TUBEROSUM, Gene Ontology, Arabidopsis thaliana, text mining methods, associative gene networks, centrality of vertices, network-based prioritization methods, база знаний SOLANUM TUBEROSUM, Gene Ontology, Arabidopsis thaliana, методы text mining, ассоциативные генные сети, центральность вершин, сетевые методы приоритизации

## Abstract

Methods for prioritizing or ranking candidate genes according to their importance based on specif ic criteria via the analysis of gene networks are widely used in biomedicine to search for genes associated with diseases and to predict biomarkers, pharmacological targets and other clinically relevant molecules. These methods have also been used in other f ields, particularly in crop production. This is largely due to the development of technologies to solve problems in marker-oriented and genomic selection, which requires knowledge of the molecular genetic mechanisms underlying the formation of agriculturally valuable traits. A new direction for the study of molecular genetic mechanisms is the prioritization of biological processes based on the analysis of associative gene networks. Associative gene networks are heterogeneous networks whose vertices can depict both molecular genetic objects (genes, proteins, metabolites, etc.) and the higher-level factors (biological processes, diseases, external environmental factors, etc.) related to regulatory, physicochemical or associative interactions. Using a previously developed method, biological processes involved in plant responses to increased cadmium content, saline stress and drought conditions were prioritized according to their degree of connection with the gene networks in the SOLANUM TUBEROSUM knowledge base. The prioritization results indicate that fundamental processes, such as gene expression, post-translational modif ications, protein degradation, programmed cell death, photosynthesis, signal transmission and stress response play important roles in the common molecular genetic mechanisms for plant response to various adverse factors. On the other hand, a group of processes related to the development of seeds (“seeding development”) was revealed to be drought specif ic, while processes associated with ion transport (“ion transport”) were included in the list of responses specif ic to salt stress and processes associated with the metabolism of lipids were found to be involved specif ically in the response to cadmium.

## Abstract (ru)

Методы приоритизации или ранжирование кандидатных генов по их важности в соответствии с заданными критериями, основанными на анализе генных сетей, широко применяются в биомедицине для поиска ассоциаций генов с заболеваниями, предсказания биомаркеров, фармакологических мишеней и т. д. При этом наблюдается тенденция их использования и в других областях знаний, в частности в растениеводстве. В зна- чительной степени это обусловлено развитием технологий для решения задач маркер-ориентированной и геномной селекции, требующих знаний о молекулярно-генетических механизмах, лежащих в основе форми- рования хозяйственно ценных признаков. Новым направлением для изучения молекулярно-генетических ме- ханизмов является приоритизация биологических процессов с применением анализа ассоциативных генных сетей. Ассоциативная генная сеть – это гетерогенная сеть, в качестве вершин которой наряду с молекулярно- генетическими объектами (гены, белки, метаболиты и т. д.) могут быть представлены сущности более высокого уровня (биологические процессы, заболевания, факторы внешней среды и т. д.), связанные между собой регу- ляторными, физико-химическими или ассоциативными взаимодействиями. С использованием разработанного нами ранее метода осуществлена приоритизация биологических процессов по степени их связи с генными се- тями, представленными в базе знаний SOLANUM TUBEROSUM и описывающими ответ растений на повышенное содержание кадмия, солевой стресс и условия засухи. Результаты приоритизации свидетельствуют о том, что фундаментальные процессы, такие как экспрессия генов, посттрансляционная модификация, деградация бел- ков, программируемая клеточная смерть, фотосинтез, передача сигналов, ответ на стресс, играют важную роль в общих молекулярно-генетических механизмах ответа растений на различные неблагоприятные факторы. С дру- гой стороны, среди специфичных для устойчивости к засухе была выявлена группа процессов, связанных с раз- витием семян (seeding development). Процессы, связанные с ионным транспортом (ion transport), вошли в список специфичных для ответа на солевой стресс, а связанные с метаболизмом липидов (phospholipid degradation – деградация фосфолипидов) – для ответа на кадмий.

## Introduction

The rapid development of high-performance experimental methods has significantly expanded the ability to generate large sets of genomic, transcriptomic and proteomic data in scientific research. This, in turn, has increased the relevance of bioinformatics methods that allow researchers to interpret omic data, both at the level of key genes and at the level of molecular genetic mechanisms. Among the widely used approaches in computational analysis of gene sets identified in experiments are prioritization methods (Raj, Sreeja, 2018), which rank the studied genes (or other objects, such as diseases) by characterising their proximity to a set from a given learning sample. Depending on the prioritization problem, the training set may consist of genes associated with diseases or phenotypic traits or sets of differentially expressed genes, for example. The higher the proximity in relative units, the greater the priority of the analysed object as a candidate possessing the same properties as objects in the training set. Such methods are used in biomedicine to detect candidate genes associated with diseases (Tranchevent et al., 2016), disease biomarkers (Jha et al., 2020), potential pharmacological targets (Cesur et al., 2020) and drug republic (Pushpakom et al., 2019). In animal husbandry and crop production, prioritization methods have been applied to analyse genomic data related to markeroriented and genomic selection (Arruda et al., 2016; Crossa et al., 2017; Kochetov et al., 2017; Kolchanov et al., 2017; Cai et al., 2019; Voss-Fels et al., 2019; Sun et al., 2020), as well as Raspanic loci analysis (Bargsten et al., 2014; Schaefer et al., 2018; Lin et al., 2019).

A special place among the prioritization methods is occupied by approaches based on the analysis of genetic network graphs, including network protein interactions, metabolic networks, signal transmission networks, and networks of genes associated with disease. In such network prioritization methods, the proximity of the studied genes to the training sample is estimated using various topological characteristics of the genetic network graph. Methods for analysing the structure of genetic network graphs to solve prioritization tasks can be divided into three large groups (Shim et al., 2017; Raj, Sreeja, 2018): (1) methods based on identifying hubs using centrality indicators of vertices in the column (Cho et al., 2016); (2) methods based on network diffusion, including random wandering (Chen et al., 2011; Shim, Lee, 2015; Le, Pham, 2017; Lysenko et al., 2017); and (3) methods based on identifying functional modules (clusters or subnets) (Jia et al., 2011; Leung et al., 2014). All of these methods are aimed at identifying genes (or other entities) that are important for the phenotype or process being studied. The importance of information about the topology of networks in assessing the functional significance of genes was demonstrated by the example of the centrality of the vertices in a Saccharomyces cerevisiae protein–protein interactions network (Jeong et al., 2001). The authors showed that the deletion of vertices with a large number of connections in the network of protein– protein interactions was fatal more often than the deletion of other vertices.

Among the frequently used characteristics to determine the importance of the vertex in the network structure are centrality indicators, including Degree Centrality, which is the number of links this vertex has with other vertices in the network (Freeman, 1978); centrality proximity to the centre of the graph (Closeness Centrality), which is the reverse amount of the sum of the lengths of all the shortest pathways passing through the top (Sabidussi, 1966); and centrality (Betweenness Centrality), which is the number of the shortest pathways passing through the vertex (Freeman, 1977).

Previously, we proposed the concept of an associative gene network (Ivanisenko V.A. et al., 2015), an extended gene network whose vertices can represent not only molecular genetic objects (genes, proteins, metabolites, etc.), but also higherlevel factors (biological processes, phenotypic traits, diseases, factors of the external environment, etc.) related to regulatory, physical, chemical or associative interactions (Ivanisenko V.A. et al., 2015). Automatic analysis of texts of scientific publications and factual databases was used to create knowledge bases: ANDSystem, which contains associative gene networks for animals and humans (Ivanisenko V.A. et al., 2015, 2019; Ivanisenko T.V. et al., 2020), and SOLANUM TUBEROSUM (Saik et al., 2017; Ivanisenko T.V. et al., 2018), which contains associative gene networks of plants. Analysis of associative gene networks from the knowledge bases ANDSystem and SOLANUM TUBEROSUM was used to develop methods for prioritizing human genes associated with diseases (Saik et al., 2018, 2019) and the potato genes involved in breeding and the development of agriculturally meaningful traits (Demenkov et al., 2019), respectively. The prioritization method was based on the assessment of the centrality indicator according to the degree of vertices corresponding to genes in the analysed associative gene networks.

In this work, an approach is proposed to prioritize biological processes by calculating the vertex centrality indicator in the associative gene network. This work uses the example of the model plant Arabidopsis thaliana and the information about the biological processes associated with the response of plants to the unfavourable factors cadmium content, drought conditions and salt stress in the knowledge base SOLANUM TUBEROSUM.

## Materials and methods

SOLANUM TUBEROSUM (Saik et al., 2017; Ivanisenko T.V. et al., 2018) consists of three main blocks.

The first (block 1) is an automatic analysis of texts of scientific publications and factual databases that is designed to extract information about the relationship between objects using semantic–linguistic templates. To extract knowledge, ANDSystem software tools configured to the subject area under study are used (Ivanisenko V.A. et al., 2015). The setting of the subject area includes the creation of new semantic– linguistic templates that take into account the specifics of the texts of scientific publications in the field of biology of plants and crop production.

The second block is the knowledge base containing object dictionaries and information about the relationships between objects extracted in block 1 in the form of an integrated associative gene network (a graph in which the vertices correspond to objects and the ribs indicate the specified types of connections). The SOLANUM TUBEROSUM knowledge base contains dictionaries of molecular genetic objects (genes, proteins, metabolites, microRNAs), Gene Ontology biological processes (Gene Ontology Consortium, 2019), phenotypic traits, diseases and pathogens of potatoes and model organisms, among other information. To describe relationships between these objects in associative gene networks, more than 25 different types of interactions are used. These correspond to, for example, physical interactions, catalytic reactions, regulation, participation and associations.

In block 3, ANDVisio (Demenkov et al., 2012), designed to reconstruct and analyse associative gene networks related to the problem under study, is applied based on the knowledge base of SOLANUM TUBEROSUM. The SOLANUM TUBEROSUM knowledge base can be accessed via the Internet athttps://www-bionet.sscc.ru/and/plant/

The number of interactions in the SOLANUM TUBEROSUM knowledge base for three plant species is shown in Table 1.

**Table 1.**
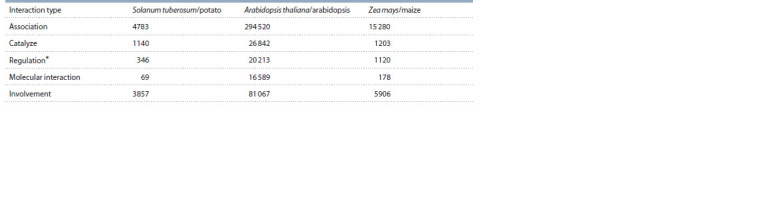
Number of basic types of interactions, including molecular genetic interactions and associations, presented in the knowledge base of SOLANUM TUBEROSUM for three plant species The total number of links is shown for the following types of interactions: upregulation, downregulation, regulation, activity upregulation, activity downregulation, activity regulation, expression upregulation, expression downregulation, expression regulation, transport upregulation, transport downregulation and transport regulation.

**Fig. 1.**
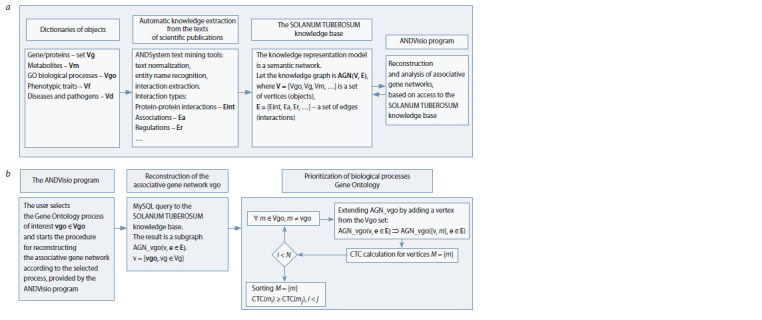
Schematic diagram of all steps performed during prioritization: a, creating the SOLANUM TUBEROSUM knowledge base; b, prioritization of Gene Ontology biological processes (Gene Ontology Consortium, 2019).

The schematic diagram of all stages of the prioritization is shown in Fig. 1. A detailed description of the development of the SOLANUM TUBEROSUM knowledge base (see Fig. 1, a) can be found in previous studies (Saik et al., 2017; Ivanisenko T.V. et al., 2018). The prioritization algorithm consists of several steps (see Fig. 1, b), which are fully automated in the ANDVisio program. The ANDVisio program provides the user interface for access to the SOLANUM TUBEROSUM knowledge base, and has a wide range of tools for reconstruction, graphic visualisation and analysis of associative gene networks. 

In the first step, the associative gene network for the problem being studied is automatically reconstructed as specified by the user, incorporating biological processes from the Gene Ontology database. To achieve this, the “Reconstruction of the Network” procedure is implemented in ANDVisio. In the interface window for this procedure, the name of the Gene Ontology biological process is selected from the list provided (which contains all of the Gene Ontology biological processes, updated annually). Upon the execution of this procedure, an associative gene network related to a given biological process will be automatically reconstructed and visualised in graphical form. The reconstruction algorithm includes an appeal using MySQL requests to the SOLANUM TUBEROSUM knowledge base, which generates a list of genes with a connection to the specified biological process. The user can choose whether to use interactions identified by analysing the texts of scientific publications or those extracted from factual databases. Genes from this list become vertices in the desired associative gene network. The formal description of the algorithm is shown in Fig. 1, b. Using this algorithm, networks were reconstructed for three biological processes: “Response to Cadmium Ion”, “Drought Tolerance” and “Response to Salt Stress”.

In the second step, for each reconstructed associative gene network, biological processes were prioritized according to the algorithm shown in Fig. 1, b. Prioritization is an automated iterative process performed by the ANDVisio program. Briefly, all Gene Ontology biological processes in the SOLANUM TUBEROSUM knowledge base dictionaries are sequentially analysed. The analysis included the calculation of the CTC (cross-talk centrality) indicator. The biological processes were ranked according to their CTC values thus, biological processes with the highest indicator received maximum priority.

The CTC indicator for the vertex i was calculated using the formula:

**Formula. 1.**
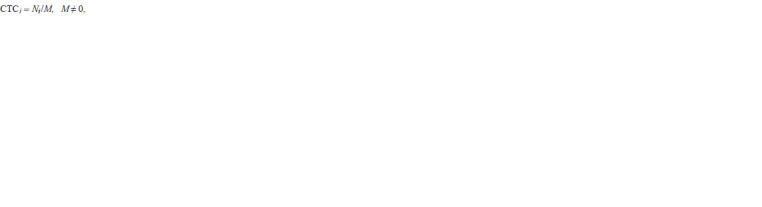
Formula. 1.

where Ni is the number of vertices to which the i vertex is connected by an edge in an analysed column of an associative gene network, M is the total number of vertices of the associative gene network, and i takes values from 1 to M. The values of the CTC indicator range from 0 to 1, with the value of 0 occurring when the peak under consideration on the network is not related to other vertices. The characteristic Ni is known as the indicator of the Degree centrality of the vertex (Freeman, 1978). This indicator is used because it is assumed that the larger the vertices associated with this vertex, the greater its influence in the associative gene network and the molecular genetic mechanism as a whole. To assess the significance of the centrality of the vertex (biological process), an approach to calculate the overrepresentation of biological processes was used (Subramanian et al., 2005). Using this approach, the probability (p-value) that this vertex will have Ni or more links by random chance in the associative gene network was evaluated using a hypergeometric distribution adjusted to the Benjamini–Yekutieli multiple comparison procedure (Benjamini, Yekutieli, 2001).

For the characterisation of the identified processes, a cluster analysis was carried out by the Markov Cluster algorithm (Van Dongen, Abreu-Goodger, 2012), using the semantic metric for biological processes by Wang and colleagues (Wang J.Z. et al., 2007).

## Results and discussion

Using the SOLANUM TUBEROSUM knowledge base, we reconstructed the associative gene networks of A. thaliana that described the interaction of genes with biological processes related to plant responses to adverse environmental factors, including drought, salt stress and increased cadmium content. To study the potential molecular genetic mechanisms underlying the reconstructed gene networks, biological processes were prioritized based on the centrality of their interactions with the network’s genes/proteins.

Plant responses to drought

**Fig. 2.**
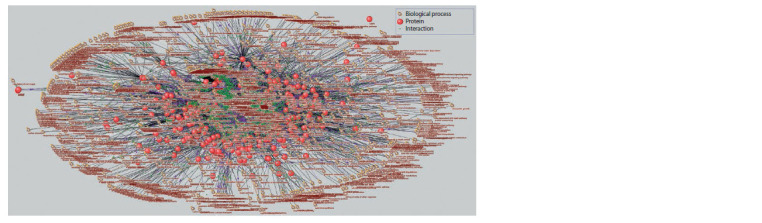
Associative gene network “drought tolerance” for Arabidopsis thaliana, including proteins and biological processes as vertices.

The associative gene network for the biological process “drought tolerance”, reconstructed in the SOLANUM TUBEROSUM knowledge base, included 292 vertices (proteins) interconnected by 440 ribs (Fig. 2). To simplify the representation of the interactions, the genes corresponding to the proteins are not illustrated. The relatively large number of proteins associated with the term “drought tolerance” can be explained by the fact that plant resistance to drought is conditioned by numerous aspects of plant physiology – a search query in PubMed for the keywords “drought” and “plants” yields more than 18,000 publications.

It should be noted that the analysis based on automatic processing of scientific literature data depends on how deeply the subject area has been studied. Thus, our conclusions can indicate the presence of an interaction, according to the published data, but cannot assert the absence of an interaction simply because the interaction is not discussed in the literature.

**Fig. 3.**
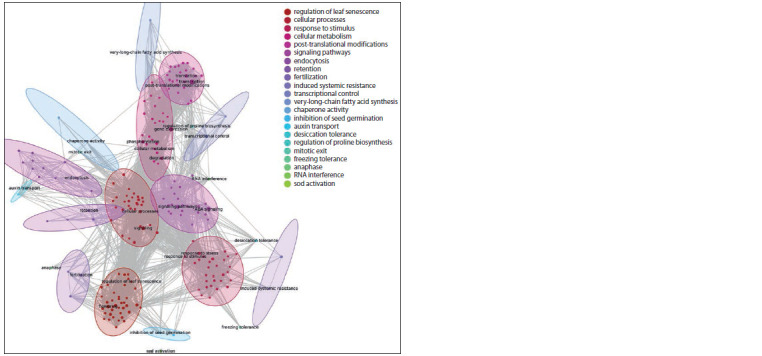
Semantic proximity clustering of biological processes signif icantly overrepresented in the “drought tolerance” associative gene network. Ovals outline vertices from one cluster. The colour of the vertices and ovals matches the colour of the cluster in the legend.

Among the biological processes associated with proteins in the associative gene network “drought tolerance”, there were 208 processes with a Q-value below the significance level of 0.05. Their centrality scores ranged from 0.0067 to 0.58. The identified biological processes were divided into 22 clusters according to semantic proximity (Fig. 3).

A list of 12 biological processes with the largest number of links with other objects in the drought tolerance gene network is shown in Table 2.

**Table 2.**
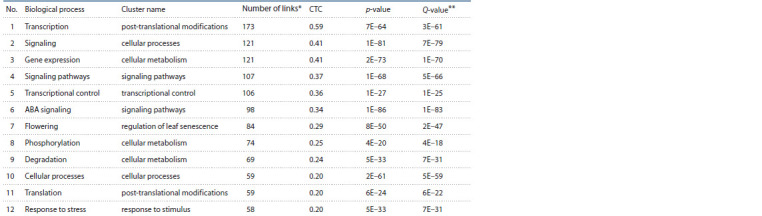
Ranking of biological processes according to their potential relationship with the “drought tolerance” process * The number of connections between the biological process and proteins/genes in the associative network. ** Signif icance adjusted for multiple Benjamini–Yekutieli comparisons.

The prioritization enables the identification of such fundamental processes as “transcription”, “signaling” and “gene expression”, which fell into the clusters “post-translational modifications”, “cellular processes” and “cellular metabolism”, respectively. The processes associated with transcription, translation and gene expression are widely discussed inthe context of transcription factors and their participation in plant responses to adverse environmental conditions (Leng, Zhao, 2020). 

Signaling processes induced by external and endogenous factors also play an important role. In particular, the process “ABA signaling” (abscisic acid signaling pathway) was highly rated (see Table 2). The CTC level for this process was found to be 0.34. This means that when the analysed associative gene network “drought tolerance” is expanded by adding the “ABA signaling” vertex, 34 % of all vertices will be associated with it. Many studies have demonstrated the high importance of ABA in plant responses to drought (approximately 3000 publications in PubMed). It has been shown that ABA is synthesised in leaves in response to a water deficiency signal transmitted from roots to shoots and is involved in mechanisms of drought resistance, such as stomata closure and production of osmotic defence proteins (Takahashi et al., 2018). ABA is also involved in the regulation of flowering time (“flowering” is on the list of priority processes, see Table 2). For example, it was shown that under drought conditions, ABA-responsive element binding factor 3 (ABF3) and ABF4, along with nuclear transcription factor Y subunit gamma (NF-YC), enhance the expression of suppressor of overexpression of constans 1 (SOC1), thus promoting acceleration of plant flowering, allowing the plant to complete its life cycle at an earlier date (Hwang et al., 2019).

Another process associated with the response of plants to drought, located in the upper lines of the prioritization results (see Table 2), is phosphorylation (one of the main ways to transmit regulatory signals in a cell). For example, it was shown in A. thaliana that the transcriptional activation of genes sensitive to drought requires phosphorylation of the transcription factor RD26 by the kinase brassinosteroid insensitive 2 (BIN2) (Jiang et al., 2019). In a study of a droughtresistant rapeseed cultivar (Brassica napus L.), an important role was shown for beta carbonic anhydrase 1 (βCA1) phosphorylation in photosynthesis regulation under drought conditions (Wang L. et al., 2016).

The position of biological processes such as plant resistance to cold and salt stress in the list of prioritized processes in the analysis of plant response to drought conditions deserves special discussion. Although these processes were not at the top of the list of processes with the highest priority for the associative gene network “drought tolerance” (see Table 2), they had a rather high rating (CTC = 0.1). Indeed, plant resistance to various adverse environmental factors is often mediated by the same molecular genetic mechanisms. For example, it was shown in corn (Zea mays) that the expression of the transcription factor MYB (MYB3R) is induced during both drought and salt stress, contributing to plant resistance to these environmental factors (Wu et al., 2019). In soybean (Glycine max), it was shown that another transcription factor in the MYB family, MYB118, also plays an important role in plant resistance to drought (Du et al., 2018). Its expression, like that of MYB3R, is induced under drought and salt stress, and the newly synthesised MYB118 enhances the expression of stress-associated genes that mediate the plant response to these stresses. In cotton (Gossypium spp.), an important role was shown for proteins in the cyclin-dependent kinase family in plant response to drought and salt stress (Magwanga et al., 2018). The mechanisms of plant response to drought overlap with the mechanisms of response to cold stress: in both cases, the synthesis and mobilisation of abscisic acid is induced in the vascular tissue of the leaves, which is necessary for the regulation of stomatal closure (reviewed in Agurla et al., 2018).

Plant response to salinity

**Fig. 4.**
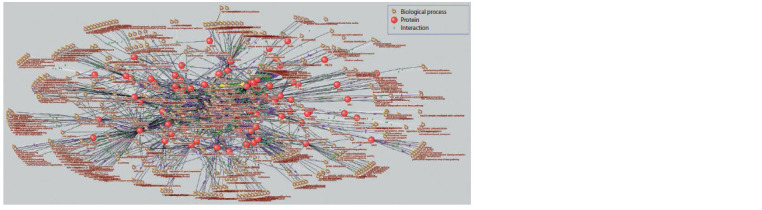
Associative gene network “response to salt stress” for Arabidopsis thaliana, including proteins and biological processes as vertices.

The “response to salt stress” associative gene network contained 81 vertices and 102 links (Fig. 4). Twelve of the highestpriority processes with a centrality index of at least 0.2 were identified (Table 3). As in the case of the drought resistance associative gene network, all of the processes included in the list of the highest priority processes are statistically significant.

**Table 3.**
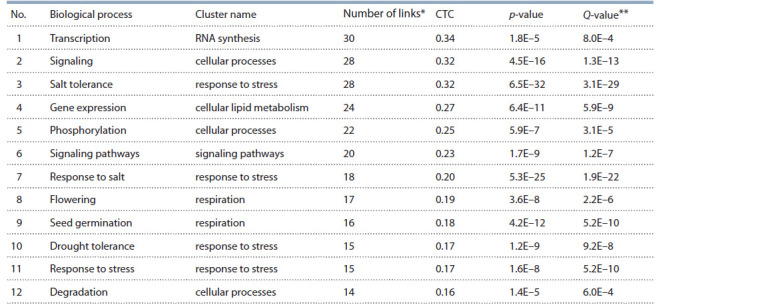
Ranking of biological processes according to their potential relationship with the “response to salt stress” process * The number of connections between the biological process and proteins/genes in the associative network. ** Signif icance adjusted for multiple Benjamini–Yekutieli comparisons.

**Fig. 5.**
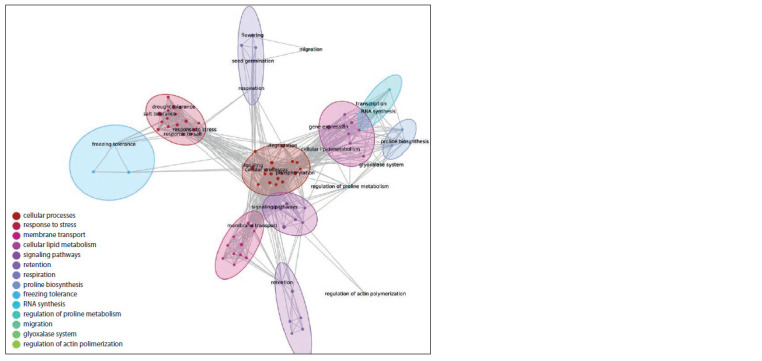
Semantic proximity clustering of biological processes signif icantly overrepresented in the “drought tolerance” associative gene network. Ovals outline vertices from one cluster. The colour of the vertices and ovals matches the colour of the cluster in the legend.

Among the biological processes associated with proteins in the “response to salt stress” associative gene network, 85 had a Q-value of < 0.05. Their centrality scores ranged from 0.02 to 0.34. The identified biological processes were divided into 14 clusters according to semantic proximity (Fig. 5).

A list of the 12 biological processes with the largest number of connections with other objects in the “response to salt stress” gene network is shown in Table 3.

Salt stress tolerance is widely represented in the scientific literature – a PubMed search for the keywords “salt” and “plants” yields more than 24,000 publications. The analysis yielded a list of top-rated processes that included “seed germination”, which fell into the “respiration” cluster (see Table 3). For example, the authors showed in Stylosanthes humilis that increased salt content suppresses seed germination and results in decreased ethylene production and increased abscisic acid production (Silva et al., 2018). Leaf senescence during normal plant development and caused by salt stress occurs via common hydrogen peroxide-mediated signaling pathways, as shown in A. thaliana (Allu et al., 2014)

Plant response to the presence of cadmium

**Fig. 6.**
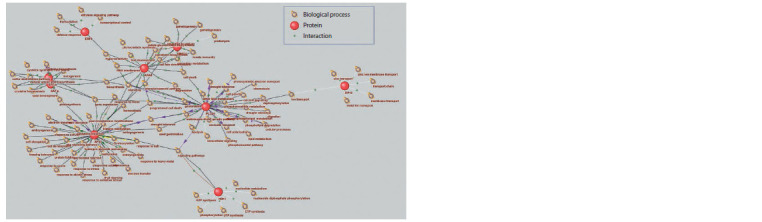
Associative gene network “response to cadmium ion” for Arabidopsis thaliana, including proteins and biological processes as vertices.

Cadmium is toxic to most plants and animals (Genchi et al., 2020). It can be present in soil, causing stress responses in plants, suppressing the growth of roots and shoots and reducing the rate of photosynthesis and nutrient consumption (Genchi et al., 2020; Kaya et al., 2020). The increased interest in the problem is due to some plants being prone to cadmium hyperaccumulation, which raises prospects for their use for both soil cleaning and other industrial purposes (Küpper, Leitenmaier, 2013). However, the mechanisms underlying plant response to cadmium remain poorly understood. Nine genes were associated with the biological process “response to cadmium ion” in the SOLANUM TUBEROSUM knowledge base for A. thaliana (Fig. 6).

**Fig. 7.**
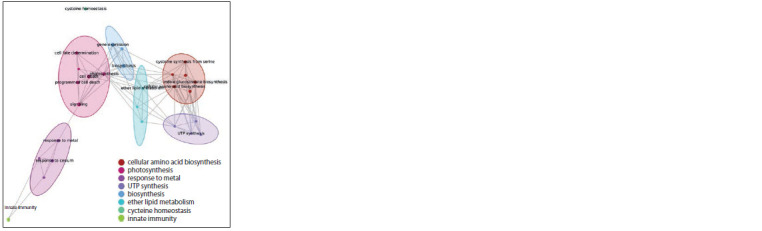
Semantic proximity clustering of biological processes signif icantly overrepresented in the “response to cadmium ion” associative gene network. Ovals outline vertices from one cluster. The colour of the vertices and ovals matches the colour of the cluster in the legend.

Among the biological processes related to proteins in the “response to cadmium ion” associative gene network, 28 had aQ-value below 0.05. Their centrality scores ranged from 0.11 to 0.44. The identified biological processes were divided into 8 clusters according to semantic proximity (Fig. 7).

A list of the 12 biological processes with the largest number of connections with other objects in the “response to cadmium ion” gene network is shown in Table 4. Among the biological processes with a high rating in the cadmium response gene network, there were two processes associated with cell death (“programmed cell death” and “cell death”), which were in the “photosynthesis” cluster. The importance of programmed cell death in response to cadmium has been confirmed in dozens of publications. For example, it was shown that cadmium causes morphophysiological changes and programmed cell death in Genipa americana L. (Souza et al., 2011); moreover, in tomato cell culture, it was demonstrated that cadmium induces programmed cell death using caspase-like proteases (Iakimova et al., 2008).

**Table 4.**
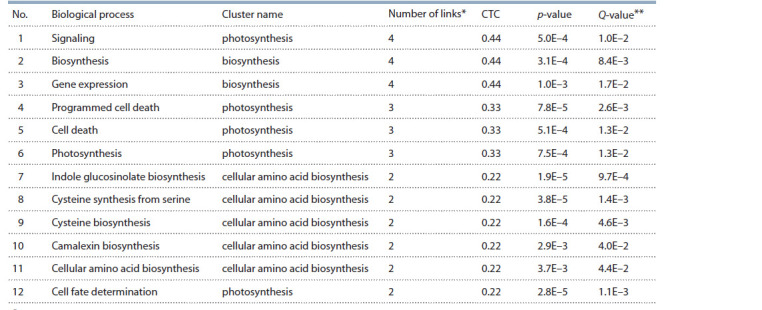
Ranking of biological processes according to their potential relationship with the “response to cadmium ion” process

In studies of the molecular mechanisms of plant responses to unfavourable factors, it may be of great interest to identify both general and specific biological processes. Below we consider examples of general and specific processes.

Comparative analysis of reconstructed associative gene networks

Comparison of the “response to salt stress” and “drought tolerance” associative networks revealed 41 common biological processes, 40 processes specific to the response to salt stress network and 156 processes specific to the drought tolerance network. Examination of all three associative gene networks revealed five common biological processes: signaling, cell death, programmed cell death, gene expression and photosynthesis. Notably, the tables of biological processes with the greatest number of connections with other objects (see Tables 2–4) mainly include general processes that have a statistically significant relationship with two or all three of the gene networks of plant responses to unfavourable factors.

Identification of biological processes specific to the plant response to drought compared with the responses to salinity and cadmium has revealed a group of processes associated with seed development: “seeding development”, “seed development”, “seed dormancy”, “seed maturation”, “regulation of seed size” and “inhibition of seed germination”. Indeed, many articles demonstrating the impact of drought on these processes have been published. It was shown that a lack of water leads to a decrease in the rate and duration of maturation of lentil seeds, leading to a decrease in their size (Sehgal et al., 2019). Similar studies on soybean lines clearly showed that drought conditions affect the quality of seed preparation and subsequent germination (Wijewardana et al., 2019).

When identifying biological processes specific to the response of plants to salinity, a group of processes associated with ion transport was distinguished: “ion transport”, “membrane transport”, “sodium transport”, “cation transport”, “transmembrane proton transport”, “sodium ion transmembrane transport” and “potassium ion homeostasis”. Indeed, the adaptation of plants to conditions of salt excess depends on their ability to remove Na+ and Cl– ions or to increase resistance to osmotic stress and ion accumulation in tissues (Munns, Tester, 2008).

Comparing the biological processes specific to the plant response to the presence of cadmium with those involved in responses to drought and salinity highlights several processes associated with lipid metabolism: “ether lipid metabolism”, “phospholipid degradation” and “glycerophospholipid metabolism”. It is known that lipid peroxidation is one of the manifestations of the toxicity of cadmium. For example, in tomatoes, it has been shown that cadmium induces substantial changes in lipid composition, causing premature aging of leaves (Djebali et al., 2005).

## Conclusion

The molecular genetic mechanisms of potato resistance to unfavourable conditions remain rather poorly understood. The accumulated knowledge about the A. thaliana model plant can shed light on the molecular interactions in the gene networks involved in the response of potatoes to stress conditions. This approach is also used in studies by other researchers. For example, Ž. Ramšak and colleagues (2018) built a model of immune signaling pathways in A. thaliana, then superimposed the data obtained onto S. tuberosum to gain new knowledge about the immune signaling pathways in potatoes.

The Gene Ontology database (Gene Ontology Consortium, 2019) contains information on biological processes and the genes involved in their functioning. Using Gene Ontology terms, molecular genetic mechanisms involved in the breeding and important traits of plants, knowledge of which is necessary for the development of modern approaches for markeroriented and genomic selection, can be described. However, the analysis of gene networks built by analysing literature sources containing data on the relationship between genes and the studied processes is also of great interest.

Analysis of the prioritization of biological processes that may be involved in the response of plants to unfavourable factors (cadmium, salinity and drought conditions), performed using the reconstruction of associative genes, showed good agreement with the well-known literature data. Among the biological processes that received high priority were fundamental processes related to the expression of genes, post-translational modification of proteins and degradation, as well as processes associated with cell death. The various ways to transmit signals occupies an important role in the mechanisms of plants’ responses to adverse factors.

When comparing the biological processes identified as playing a role in responses to unfavourable conditions, in addition to finding common processes associated with all three of the unfavourable factors in the external environment, we also identified processes that were statistically significantly connected with only one of the associative networks studied. Among the biological processes specifically involved in response to drought is a rather large group associated with the development of seeds (for example, “seeding development” and “seed dormancy”). Among the processes involved specifically in the response to saline stress are a group of processes associated with ion transport (for example, “ion transport”, “transmembrane proton transport” and “sodium ion transmembrane transport”). Finally, processes involved in lipid metabolism (such as “phospholipid degradation”) were specific to the plant response to cadmium.

## Conflict of interest

The authors declare no conflict of interest.
